# Social conditioning and extinction paradigm: a translational study in virtual reality

**DOI:** 10.3389/fpsyg.2015.00400

**Published:** 2015-04-07

**Authors:** Youssef Shiban, Jonas Reichenberger, Inga D. Neumann, Andreas Mühlberger

**Affiliations:** ^1^Department of Clinical Psychology and Psychotherapy, Institute of Psychology, University of Regensburg, Regensburg, Germany; ^2^Department of Behavioral and Molecular Neurobiology, Institute of Zoology, University of Regensburg, Regensburg, Germany

**Keywords:** social fear conditioning, virtual reality, operant conditioning paradigm, heart rate, fear-potentiated startle, social anxiety

## Abstract

In human beings, experiments investigating fear conditioning with social stimuli are rare. The current study aims at translating an animal model for social fear conditioning (SFC) to a human sample using an operant SFC paradigm in virtual reality. Forty participants actively (using a joystick) approached virtual male agents that served as conditioned stimuli (CS). During the acquisition phase, unconditioned stimuli (US), a combination of an air blast (5 bar, 10 ms) and a female scream (95 dB, 40 ms), were presented when participants reached a defined proximity to the agent with a contingency of 75% for CS+ agents and never for CS– agents. During the extinction and the test phases, no US was delivered. Outcome variables were pleasantness ratings and physiological reactions in heart rate (HR) and fear-potentiated startle. Additionally, the influence of social anxiety, which was measured with the Social Phobia Inventory scale, was evaluated. As expected after the acquisition phase the CS+ was rated clearly less pleasant than the CS–. This difference vanished during extinction. Furthermore, the HR remained high for the CS+, while the HR for the CS– was clearly lower after than before the acquisition. Furthermore, a clear difference between CS+ and CS– after the acquisition indicated successful conditioning on this translational measure. Contrariwise no CS+/CS– differences were observed in the physiological variables during extinction. Importantly, at the generalization test, higher socially fearful participants rated pleasantness of all agents as low whereas the lower socially fearful participants rated pleasantness as low only for the CS+. SFC was successfully induced and extinguished confirming operant conditioning in this SFC paradigm. These findings suggest that the paradigm is suitable to expand the knowledge about the learning and unlearning of social fears. Further studies should investigate the operant mechanisms of development and treatment of social anxiety disorder.

## Introduction

Social anxiety disorder (SAD) is a very common anxiety disorder, which is characterized by intense anxiety while facing social interactions, e.g., being observed or judged by other individuals. SAD has a lifetime prevalence of 12–13% among unconditioned stimuli (US) population (Kessler et al., 2005; Bandelow and Wedekind, 2014), a median lifetime prevalence of 7% in Europe (Fehm et al., 2005) and a point prevalence of 4% (Ohayon and Schatzberg, 2010). In addition to acute symptoms of distress, avoidance behavior is commonly observed in SAD patients, which has destructive consequences for social and occupational functioning (Ollendick and Hirshfeld-Becker, 2002; Stein and Stein, 2008; American Psychiatric Association, 2013; Bandelow and Wedekind, 2014).

Currently, cognitive-behavioral therapy (CBT) shows effective outcomes in SAD. The goal of the CBT is to acquire skills to identify, interrupt, and correct dysfunctional assumptions in order to develop behavior more adapted to social situations, e.g., no unrealistic fear of being judged by others negatively. The CBT also uses repeated exposure to feared situations in order to reduce the fear responses (Stangier et al., 2003; Powers et al., 2008; Bandelow and Wedekind, 2014; Craske et al., 2014). However, the efficacy of CBT is not fully satisfactory and leaves a substantial number of non-responders (Fedoroff and Taylor, 2001; Craske et al., 2014). To maximize the impact of psychotherapeutic interventions it is essential to elucidate the mechanisms underlying the etiology of SAD, which often include conditioning processes (Ollendick and Hirshfeld-Becker, 2002; Mineka and Zinbarg, 2006; Mineka and Oehlberg, 2008). Recently, new empirical data regarding fear conditioning in a non-social as well as social context have been gained using rodent models of cued fear conditioning (Toth et al., 2012) and social fear conditioning (SFC), respectively, in male mice and rats (Toth and Neumann, 2013). Toth et al. (2012) utilized the SFC paradigm by associating naturally occurring social preference behavior of male mice toward an unfamiliar conspecific (Lukas and Neumann, 2012) with an aversive event (1 s electric foot shock, 0.7 mA). During social fear acquisition, male mice were conditioned to associate the shock-induced pain with the exploration of the unfamiliar conspecific (social stimulus) and consequently, showed specific (and long-lasting) avoidance and fear of the social stimulus. During social fear extinction performed on the following day, SFC mice were repeatedly exposed to different conspecifics in their home cage without foot-shock, which resulted in an extinction of social fear and a reversal of social preference behavior. Thus the SFC paradigm has been proven useful for the investigation of neurobiological mechanisms related to social fear in animals (Toth et al., 2012; Toth and Neumann, 2013; Zoicas et al., 2014).

However, studies investigating SFC in humans are more complicated due to the complex human social–cultural circumstances and the difficulty to establish controlled experimental conditions of social interactions. Therefore, this phenomenon has been rarely investigated (Delgado et al., 2006). Traditional laboratory paradigms that model social situations by, e.g., presenting pictures of faces (e.g., Lissek et al., 2008) are criticized for insufficient external (ecological) validity (Blascovich et al., 2002). On the other hand, investigating social interactions by including real persons (e.g., associates of the experimenter) is associated with higher costs, logistical efforts, and leads to uncontrolled aspects of the paradigm since the behavior of these persons could not be fully controlled (Blascovich et al., 2002). One step to overcome these problems is to use video clips (Weiss et al., 2004) or animated faces (Mühlberger et al., 2008; Weyers et al., 2009; Schwarz et al., 2013). Another step further is to apply virtual reality (VR). Investigating social interactions in VR has several advantages: confounding variables can be controlled, environment variables can be manipulated in a multimodal manner, social situations can easily be standardized, active social behavior could be measured, and the technique is also economical (Blascovich et al., 2002; Bohil et al., 2011).

Virtual reality is designed to computer-generate a feeling of being “present” in the virtual situation by giving immersive sensory perceptions and allow interaction with the virtual environment. The feeling of presence might be a crucial factor to investigate social interactions and social fear as it is relevant for real live situations. The causal relationship between presence and fear is a matter of debate (Bouchard et al., 2008), actual aspects are discussed in a recent review (Diemer et al., 2015).

The literature shows that although the quality of the simulation is not yet comparable to real-life situations, VR offers an approach to simulate the complexity of real-world experiences in a laboratory environment in the context of phobias (Mühlberger et al., 2006, 2007; Powers et al., 2008; Shiban et al., 2013). Furthermore, the capability of VR to model acquisition, extinction, spontaneous recovery, and generalization of fear has been extensively shown for classical conditioning of non-social stimuli (e.g., Ewald et al., 2014; Glotzbach et al., 2012; Mühlberger et al., 2014; for an overview, see Glotzbach-Schoon et al., 2013).

Dunsmoor et al. (2014) published the first study investigating conditioning with social stimuli in VR with a human sample. The authors used two 3D characters (agents) in different VR environments for a SFC experiment on two consecutive days and investigated the properties of extinction in single and multiple contexts. Participants were moved passively to the two different agents in each phase. Dunsmoor et al. (2014) showed that fear acquisition and fear extinction were successful according to their primary dependent measure—fear-potentiated startle—by using electric shocks (to the participant’s wrist) as US. Spontaneous recovery, which was tested in the extinction context, was evident in both single and multiple extinction contexts. Reinstatement, which was tested in a novel context, was only observed in groups with single extinction context.

The aim of the present study was to establish an operant SFC paradigm for human studies and therefore we translated the SFC paradigm established by Toth et al. (2012) in rodents using social stimuli (agents) in VR via head-mounted-display (HMD). In a first step we investigated basic SFC mechanisms in healthy participants. Furthermore, we examined if social anxiety influences the affective learning in the SFC paradigm. Fear response was measured on a self-report level (pleasantness ratings) as well as on a physiological level [heart rate (HR), fear-potentiated startle]. Using VR 3D virtual human agents enable us to simulate a social interaction that is more difficult to implement in a PC setup. Furthermore, the use of movement in the VR was highly useful in terms of ecological validity, since one of our goals was to translate the study from Toth et al. (2012). A further advantage in using HMD is that we can ensure that the participant’s attention is focused on the experiment, as the participant can only see the VR environment with the agent in it and is not distracted by the laboratory and the examiner.

## Materials and Methods

### Participants

Forty-six healthy volunteers were recruited through advertisement at the University of Regensburg. Exclusion criteria were age below 18 or above 55, current psychiatric or psychological treatment, history of psychotropic drug use, color blindness, and defective hearing. These criteria were assessed via a questionnaire after the written informed consent was obtained. Forty participants were included (92.5% female, aged between 18 and 44, *M* = 22.0, SD = 5.22). All were students at the University of Regensburg. As compensation, they were offered credit points. All of the participants had normal or corrected-to-normal vision. Six participants were excluded due to a technical error during data acquisition. The Ethics Committee of the University of Regensburg approved the study.

### Materials

**FIGURE 1:**
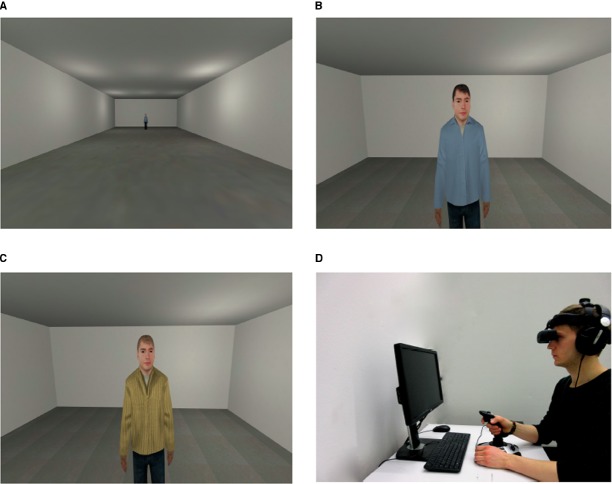
**Virtual environment. (A)** Room where acquisition and extinction phase in VR took place. **(B,C)** Two of the social stimuli (agents) used for the conditioning. **(D)** Setting (VR was presented via a head-mounted-display) during the experiment (laboratory room was darkened).

During the study participants were immersed in VR. The VR environment consisted of two rooms (see Figure 1A). In the first room, the rating room (RR), the participant’s ability to move was turned off and the upper bodies of four male virtual agents (see Figures 1B,C) appeared successively. In VR agents could be integrated in different ways, either by (animated) 3D computer-generated models, or by integrating video clips in the virtual environment. The advantage of video clips is the photorealistic visual presentation. However, the advantage of computer-generated agents is the possibility to integrate online-interactive behavior, allow a free movement of the participant and to view the agent correctly in a 3D manner from different directions. The agent gazed dynamically at the participant and moved his head and upper body slightly to appear alive. The second room was similar to the first room, but had more depth and was used for the learning procedure (conditioning, extinction, spontaneous recovery test). The participant was positioned at one end of the room and could see the agent at the other end of the room. When the participant reached the agent (as described below) in some of the trials aversive consequences followed. Aversive consequences consisted of an air blast toward the right side of the participant’s neck (5 bar, 10 ms) and a female scream (95 dB, 40 ms; see Schmitz and Grillon, 2012). Both stimuli were administered simultaneously. A compressed tank of air was regulated via a magnetic valve system and channeled through a tube, which was fixed to the participant’s torso.

The VR was presented to participants via a Z800 3D Visor HMD (eMagin, NY, USA) and was generated via Steam Source engine (Valve Corporation, Bellevue, WA, USA). “Cybersession” software (VTplus GmbH, Würzburg, Germany) controlled the presented VR environment (see Figure 1D). The participant’s head position was monitored via the Patriot electromagnetic tracking device (Polhemus Corporation, Colchester, VT, USA) which adjusts the field of view (FOV) to head movements. Sounds were presented over headphones (Sennheiser HD-215, Sennheiser electronic GmbH, Germany). Participants used a joystick (Logitech Extreme 3D Pro Joystick, Logitech GmbH, Germany) to move in the VR environment. Physiological data were monitored, and digitally amplified (V-Amp, Brain Products GmbH, Germany) and recorded (Brain Vision Recorder software, Version 1.20, Brain Products GmbH, Germany).

### Measures

Participants filled in a demographic questionnaire (age, sex, education, and current occupation) and the Social Phobia Inventory (SPIN; Connor, 2000; German version: Stangier and Steffens, 2002) to assess social fear.

The SPIN consists of 17 items that assess fear, avoidance, and physiological symptoms of social phobia in the previous week. Answers are given on a 5-point Likert scale (from 0 = “not at all” to 4 = “extremely”). The German version of the SPIN was evaluated by Sosic et al. (2008). Internal consistency (Cronbach’s alpha) was excellent with 0.95 for a representative sample of 2043 Germans. Convergent and divergent validity are satisfactory. Furthermore, the German version of the SPIN is a sensitive and specific measure for social phobia as it distinguishes successfully between social phobia and other mental disorders (Sosic et al., 2008).

In order to measure the experienced pleasantness of the agents, ratings were asked verbally during the presentations of the agents in the rating room (“How pleasant/unpleasant do you feel in the presence of this agent?”). These ratings had a range from 0 (very unpleasant) to 10 (very pleasant).

Besides the subjective measures, physiological data were collected. To record the EMG (electromyography) of the musculus orbicularis as a measure of fear-potentiated startle, two surface electrodes (Ag/AgCl, Ø = 8 mm) filled with electrode cream were administered under the right eye of the participant. Reference (right) and ground (left) electrodes (Ag/AgCl, Ø = 8 mm) were placed on the mastoids. Two adhesive pre-gelled surface electrodes (Ag/AgCl, Ø = 40 mm) were attached to the middle of the upper chest and on the rib tip of the left half of the body to record the ECG (electrocardiography).

### Procedure

The experiment was conducted in two sessions taking place on two succeeding days. On day 1 the session consisted of filling out the questionnaires, the acquisition phase, a 10-min break and the extinction phase [duration on day 1 was 80 min (60 min in VR)]. The test phase followed 24 h later [duration on day 2 was 40 min (20 min in VR)].

Conditioning was conducted in eight blocks. One block consisted of two presentations of both conditioned stimuli (CS) with aversive reinforcement in terms of air blast and scream (CS+) and without aversive reinforcement (CS–), resulting in 16 presentations each per participant. The order within each block was randomized. Which pair of the four agents was presented as CS+/CS–, was balanced across participants. The CS–US contingency was set at 75 %.

The extinction phase consisted of 12 blocks that looked exactly the same as those in the acquisition phase, except there was no US. Because 12 instead of 8 blocks were presented, the number of trials was 24 in this phase. Before and after the acquisition and extinction phase, ratings were asked in the rating room, where no US was administered. In the test phase, that took place in the rating room, four known agents and four unknown agents (CS∼, differently clothed) were presented three times each.

For each rating phase in the rating room (before and after acquisition, before and after extinction, and one in the test phase) each agent was presented three times (presentation 8 s, inter-stimulus interval 20 s). Startle noise (white noise: 108 dB, 40 ms) was administered between second 6 and 8 (pseudo-randomized) in every inter-trial interval.

In the first session participants were briefed in written form and the informed consent was signed. After filling in the demographic questionnaire and the SPIN, participants were prepared for the VR part of the experiment. The electrodes, the air blast device, the HMD and the headphones were adapted. During the experiment the laboratory room was darkened and participants received recorded instructions, which were delivered via headphones. At first, participants relaxed for 2 min in VR (black screen) for a baseline-measure. Then the VR presentation started. First the four agents were presented one after another in the rating room. Now participants were asked to rate the experienced pleasantness during the presentation of each agent.

The acquisition phase started in the second room. Participants received the recorded instruction: “You will now meet virtual human beings again. Please use the joystick to approach the person. Please try to move directly toward the person. Press the joystick forward to move straightforward and approach the person.” Participants had to approach the agents actively via a joystick and as soon as they reached a specific distance to the agents (30 cm), lights faded out. At this moment, the US was presented for CS+ agents in 75% of the trials. Participants could move lateral, diagonal or away from the agent. Movement of the head caused a view change unrelated to the movement, i.e., participants could theoretically look away while moving toward the agent (we observed, however, no such behavior). After the end of the acquisition phase, participants had to rate the agents again in the rating room as described above. Subsequently a 10-min break followed during which the participants were allowed to rest and to take the HMD off.

The following extinction phase began with 2 min relaxation followed by rating the agents in the rating room. For the extinction the same procedure was applied as for the acquisition, with the only difference that no aversive stimuli (neither air blast nor scream) were presented. After participants had rated the agents for the last time in the rating room, the session on day 1 ended.

The second session took place 24 h later. Participants were prepared for the test phase in VR as described above. After a 2-min relaxation, the test phase followed. It consisted of pleasantness ratings in the rating room as described above, with the difference that not only the four known agents (CS+, CS–) were shown, but also four unknown agents (CS∼). They wore different clothes and had different hair, but had the same faces as the agents shown on the 1st day. After finishing the ratings, the participants were debriefed.

### Data Reduction and Statistical Analysis

Physiological data were preprocessed with Brain Vision Analyzer 2.0 software (Brain Products GmbH, Munich, Germany) and further analyses were performed in SPSS 22.0 (IBM Corp., Armonk, NY, USA).

For fear-potentiated startle, at first, differences between the two EMG electrodes were computed (see Blumenthal et al., 2005). Then, a 250 Hz high cut-off filter, a 30 Hz low cut-off filter, and a 50 Hz notch filter were administered, the data were rectified, and a moving average (50 ms) was calculated. For each fear-potentiated startle a baseline correction was conducted using the mean value of the 50 ms before each startle tone as baseline. Next, peaks were marked automatically and manually controlled and corrected if necessary. Finally *T*-values for the startle magnitude were calculated.

For heart rate, the different values between the ECG electrodes were computed, a 1.59 Hz (12 dB) high cut-off filter, a 30 Hz (12 dB) low cut-off filter, and a 50 Hz notch filter were administered. Then R-spikes were automatically detected and counted, manually controlled and corrected if necessary. The HR per minute was exported for 6 s following a stimulus, so it could be guaranteed, that minimum five heartbeats are included into the analysis (Prescott et al., 1992).

For each outcome variable (pleasantness ratings, HR, and fear-potentiated startle) measured in the rating rooms means for CS+, CS–, and CS∼ (novel agents, only in the test phase) were calculated. For pleasantness ratings, HR, and fear-potentiated startle a repeated-measures ANOVA with the within-subjects factor time (Pre vs. Post), stimulus (CS+ vs. CS–) and between-subjects factor anxiety (low vs. high) was applied for each phase (acquisition and extinction). Data from the test phase were analyzed with an ANOVA containing the same factors as described for the acquisition and extinction phase, but were compared to the post-extinction measures. For the generalization effect during the test phase on the 2nd day, an ANOVA with the within-subjects factor stimulus (CS+ vs. CS– vs. CS∼) and between-subjects factor anxiety (low vs. high) was applied. Participants were divided into two groups (low vs. high anxiety) via a median split (median = 15) of the SPIN score.

In additional analyses of significant effects of time, stimulus, or social anxiety Student’s *t*-tests were performed. Partial η^2^ (${\text{η}}_{\text{p}}^{\text{2}}$) scores and Cohen’s *d* were used as indices of effect size. The significance level was set at two-tailed alpha = 0.05.

## Results

### Pleasantness Ratings

**FIGURE 2:**
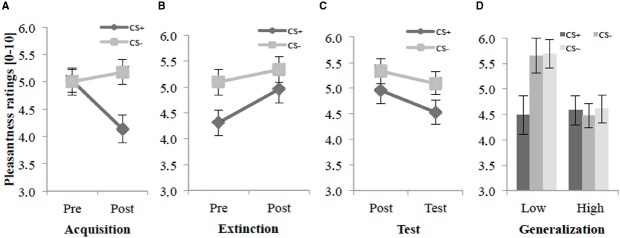
**Pleasantness ratings (*n* = 40) for CS+ and CS– pre and post acquisition (A), extinction (B), and test phase (C) as well as the generalization effect (D) for CS+, CS– and CS∼ during test phase for low and high socially fearful participants.** Note: CS+, agents with aversive unconditioned stimulus (US); CS–, agents without aversive US; CS∼, new agents on the 2nd day without aversive US; Pre, before each phase; Post, after each phase; Test, test phase. Mean pleasantness ratings (0 = least pleasant to 10 = most pleasant) were given. For the generalization effect **(D)** mean pleasantness ratings (0 = least pleasant to 10 = most pleasant) for low (Low, *n* = 21) and for high socially anxious participants (High, *n* = 19) were given. Anxiety was measured with the German version of the Social Phobia Inventory (SPIN; Stangier and Steffens, 2002), and participants were divided via median split of the SPIN (median = 15) in two groups (low and high socially anxious). Standard errors are presented by error bars.

Before the acquisition phase participants rated all agents as neutral (Figure 2A), but after the acquisition phase they reported significantly lower pleasantness for the CS+ than for the CS– agents. An ANOVA on the pleasantness ratings confirmed a significant main effect of Stimulus, *F*(1,38) = 9.73, *p* = 0.018, ${\text{η}}_{\text{p}}^{\text{2}}$ = 0.14, and significant interaction effects of Stimulus × Social Anxiety, *F*(1,38) = 4.79, *p* = 0.035, ${\text{η}}_{\text{p}}^{\text{2}}$ = 0.11, and Time × Stimulus, *F*(1,39) = 9.93, *p* < 0.003, ${\text{η}}_{\text{p}}^{\text{2}}$ = 0.20. Follow-up tests of the significant Stimulus × Social Anxiety interaction revealed a significant difference for the lower socially fearful participants (*p* < 0.002) between CS+ (*M* = 4.52, SD = 1.24) and CS– (*M* = 5.46, SD = 1.10), but not for the higher socially fearful participants. Follow-up *t*-tests of the Time × Stimulus interaction revealed no significant difference between CS+ and CS– before the acquisition (Pre), but a significant difference after the acquisition (Post), *t*(39) = –3.01, *p* < 0.005, *d* = 0.66. Furthermore, an explorative *t*-test showed a significant pleasantness decrease for the CS+ in pre- vs. post-acquisition, *t*(39) = 3.40, *p* < 0.002, *d* = 0.61. These results indicate that according to the pleasantness ratings, SFC took place.

Figure 2B shows that there was still a difference between CS+ and CS– before the extinction. After the extinction phase, CS+ and CS– agents did not vary anymore. Participants rated the CS+ after the extinction as more pleasant than before extinction. An ANOVA on the pleasantness ratings revealed significant main effects of Time, *F*(1,38) = 15.6, *p* < 0.001, ${\text{η}}_{\text{p}}^{\text{2}}$ = 0.29, Stimulus, *F*(1,38) = 5.34, *p* = 0.026, ${\text{η}}_{\text{p}}^{\text{2}}$ = 0.12, a significant interaction of Stimulus × Anxiety, *F*(1,38) = 6.22, *p* = 0.017, ${\text{η}}_{\text{p}}^{\text{2}}$ = 0.14, and a marginally significant interaction of Time × Stimulus, *F*(1,38) = 3.76, *p* = 0.060, ${\text{η}}_{\text{p}}^{\text{2}}$ = 0.09, for the extinction phase. Follow-up tests of the significant Stimulus × Anxiety interaction confirmed a significant difference (*p* < 0.001) between CS+ (*M* = 4.57, SD = 1.53) and CS– (*M* = 5.72, SD = 1.43) for the lower socially fearful participants, but not for the higher socially fearful participants. Explorative *t*-tests showed a significant difference between CS+ and CS– only pre extinction, *t*(39) = 2.45, *p* = 0.019, *d* = 0.51. Further explorative follow-up tests revealed a significant pleasantness increase for the CS+ in pre- vs. post-extinction, *t*(39) = –3.83, *p* < 0.001, *d* = 0.41, but no such effect was observed for the CS–. These results indicate that according to the ratings, social fear extinction was successfully.

Figure 2C demonstrates that in the test phase participants differentiate between CS+ and CS– and report less pleasantness for the CS+ on the 2nd day than on day 1. For the test phase (compared to post-extinction), an ANOVA on the pleasantness ratings confirmed significant main effects of Time, *F*(1,38) = 8.54, *p* = 0.006, ${\text{η}}_{\text{p}}^{\text{2}}$ = 0.18, Stimulus, *F*(1,38) = 4.95, *p* = 0.032, ${\text{η}}_{\text{p}}^{\text{2}}$ = 0.12, and a significant interaction of Stimulus × Anxiety, *F*(1,38) = 7.73, *p* = 0.008, ${\text{η}}_{\text{p}}^{\text{2}}$ = 0.17. Follow-up tests of the significant interaction effect confirmed a significant difference (*p* < 0.001) between CS+ (*M* = 4.75, SD = 1.51) and CS– (*M* = 5.74, SD = 1.36) for the lower socially fearful participants, but not for the higher socially fearful participants. Follow-up *t*-tests revealed a significant pleasantness decrease between post-extinction and test phase only for the CS+, *t*(39) = 2.75, *p* = 0.009, *d* = 0.28 for the significant main effect of Time. In terms of fear of the CS+ renewal took place.

Further explorative analyses reflect the generalization effect separately for lower and higher socially fearful participants. Figure 2D shows that during the test phase participants still distinguish between CS+ and CS–, and also distinguish between CS+ and CS∼. For the generalization effect during the test phase, an ANOVA confirmed a significant main effect of Stimulus, *F*(1.64,62.4) = 6.20, *p* = 0.006, ${\text{η}}_{\text{p}}^{\text{2}}$ = 0.14, ε = 0.82, and a significant interaction of Stimulus × Anxiety, *F*(1.64,62.4) = 7.11, *p* < 0.003, ${\text{η}}_{\text{p}}^{\text{2}}$ = 0.16, ε = 0.82. Follow-up tests on the interaction effect confirmed significant differences (both *p*s < 0.001) between CS+ (*M* = 4.48, SD = 1.70) and CS– (*M* = 5.65, SD = 1.56), and between CS+ and CS∼ (*M* = 5.68, SD = 1.28) for the lower socially fearful participants, but not for the higher socially fearful participants. These results indicate that according to the pleasantness ratings generalization could be observed for the higher socially fearful participants, but not for the lower socially fearful participants (see Figure 2D).

### Heart Rate

**FIGURE 3:**
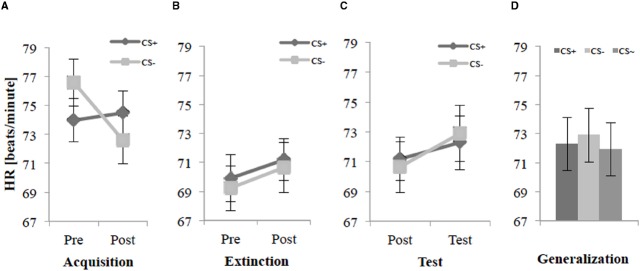
**Heart rate (*n* = 28) for CS+ and CS– pre and post acquisition (A), extinction (B), and test phase (C) as well as the generalization effect (D) for CS+, CS–, and CS∼ during test phase.** Note: CS+, agents with aversive unconditioned stimulus (US); CS–, agents without aversive US; CS∼, new agents on the 2nd day without aversive US; Pre, before each phase; Post, after each phase; Test, test phase. Mean heart rate (HR, beats/minute) was given. Standard errors are presented by error bars.

Figure 3A illustrates that the HR accelerated by a CS– agent before the acquisition, but after this phase the HR decreased for CS– while the HR for CS+ remained at the same level. For the acquisition phase, an ANOVA confirmed a significant interaction effect of Time × Stimulus, *F*(1,26) = 17.1, *p* < 0.001, ${\text{η}}_{\text{p}}^{\text{2}}$ = 0.40. Follow-up *t*-tests of the Time × Stimulus interaction revealed a significant lower HR for the CS+ than the CS– before (Pre) the acquisition, *t*(27) = –4.14, *p* < 0.001, *d* = 0.32, and a higher HR for the CS+ than the CS– after (Post) the acquisition, *t*(27) = 2.69, *p* = 0.012, *d* = 0.23 (see Figure 3A). Even that the CS– was lower than the CS+ after acquisition phase the SFC was successful according to the HR.

For the extinction phase, Figure 3B shows a lower HR for both CS+ and CS– than for the acquisition phase. An ANOVA on the HR for the extinction phase did not find any significant main or interaction effects.

For the test phase (see Figure 3C), an ANOVA revealed a significant interaction effect of Time × Stimulus, *F*(1,26) = 4.74, *p* = 0.039, ${\text{η}}_{\text{p}}^{\text{2}}$ = 0.15. Follow-up *t*-tests on this interaction found no significant difference between CS+ and CS– for post-extinction or the test phase. Further an explorative analysis reflects the generalization effect. For the generalization effect during the test phase on the 2nd day, an ANOVA showed no significant main or interaction effects. An explorative *t*-test showed only a significant difference between CS– and CS∼, *t*(27) = 3.18, *p* < 0.004, *d* = 0.10. In sum, no renewal or generalization could be observed in HR (see Figure 3D).

### Fear-Potentiated Startle

**FIGURE 4:**
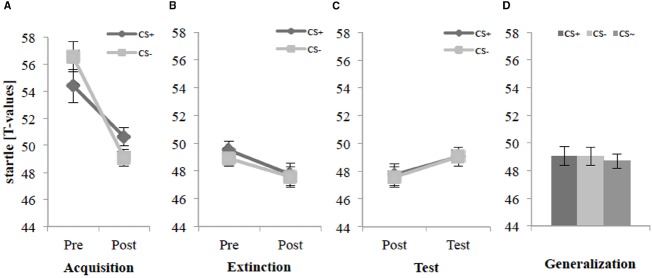
**Fear-potentiated startle (*n* = 32) for CS+ and CS– pre and post acquisition (A), extinction (B), and test phase (C) as well as the generalization effect (D) for CS+, CS–, and CS∼ during test phase.** Note: CS+, agents with aversive unconditioned stimulus (US); CS–, agents without aversive US; CS∼, new agents on the 2nd day without aversive US; Pre, before each phase; Post, after each phase; Test, test phase. Mean fear-potentiated startle (presented in *T*-values) was given. Standard errors are presented by error bars.

For the acquisition phase, an ANOVA on the fear-potentiated startle (see Figure 4A) confirmed a significant main effect of Time, *F*(1,30) = 18.4, *p* < 0.001, ${\text{η}}_{\text{p}}^{\text{2}}$ = 0.38, and a significant interaction effect of Time × Stimulus, *F*(1,30) = 9.40, *p* < 0.005, ${\text{η}}_{\text{p}}^{\text{2}}$ = 0.24. Follow-up *t*-tests of the Time × Stimulus interaction revealed no significant difference between CS+ and CS– before the acquisition phase (Pre), yet a significant difference after the acquisition phase (Post) was found, *t*(31) = 2.51, *p* = 0.018, *d* = 0.44. This indicates that according to the fear-potentiated startle, SFC was successfully. Figure 4A shows also a typical habituation for the fear-potentiated startle.

For the extinction phase (see Figure 4B), an ANOVA on the fear-potentiated startle revealed only a significant main effect of Time, *F*(1,30) = 5.22, *p* = 0.030, ${\text{η}}_{\text{p}}^{\text{2}}$ = 0.15. Follow-up test revealed no significant differences for the CS+ and CS– in pre- vs. post-extinction. For the test phase and the generalization effect (see Figures 4C,D), an ANOVA revealed no significant main or interaction effects.

## Discussion

The aim of this study was to evaluate, whether SFC can be achieved in an operant conditioning paradigm in VR. To this end, we utilized an operant SFC paradigm, where participants actively approached social stimuli in form of virtual male agents using a joystick. Acquisition, extinction, return, and generalization of social fear were operationalized via pleasantness ratings and physiological measures (HR and fear-potentiated startle).

In the operant conditioning paradigm, fear acquisition was successful both according to the subjective ratings and physiological measures. For the ratings, there was a clear decrease in the pleasantness for the CS+ compared to the CS– post acquisition. Furthermore, the HR remained high for the CS+ after the acquisition, while the HR for the CS– clearly decreased. The fear-potentiated startle data reflects that the reaction to the CS– clearly attenuated stronger than to the CS+.

Fear extinction was evident in the ratings. The difference in pleasantness between CS+ and CS– that followed acquisition vanished during extinction. Extinction effects were not statistically significant on the physiological variables. Possible reasons are that the HR and fear-potentiated startle were already too low at the beginning of the extinction phase due to fast extinction.

According to our data SFC could be induced and extinguished conforming to the operant conditioning paradigm. Further, spontaneous recovery of fear conditioning for the CS+ could be clearly observed only for the pleasantness ratings on the succeeding day. Interestingly, whereas lower socially fearful participants differentially evaluated the CS+ from the CS– and CS∼ on the 2nd day and only rated the CS+ as unpleasant, higher socially fearful participants rated all three different agent stimuli on an similar level of unpleasantness. So we found a generalization effect between CS+, CS–, and CS∼ for the higher socially fearful participants. No generalization effect between CS+, CS–, and CS∼ was found for the physiological measures.

Our results are in line with the study of Dunsmoor et al. (2014). The single context group in the study from Dunsmoor et al. (2014), described in the introduction, has similarities to our experimental setting, but also significant differences. Unlike us, they used electric shocks as US, which could have a more intense effect than an air blast and scream. However, they only measured the fear-potentiated startle, while we measured additionally the subjective pleasantness and the HR. Additionally, they only presented one agent as CS+ and one as CS–, what possibly facilitated the differentiation between the CS+ and CS–. Furthermore, they utilized four different 3D environments that varied in color and texture, because of their study goal of investigating the properties of extinction in single and multiple contexts. Importantly, our participants were conditioned using an operant conditioning procedure, i.e., they could actively move to the agents using a joystick, in contrast, participants were moved passively in VR in the experiment of Dunsmoor et al. (2014). Thus, the findings in Dunsmoor et al. (2014) and our study show that conditioning mechanisms work for social stimuli in healthy humans using a VR environment. Social fear is rarely learned passively, because people usually behave actively in social situations, e.g., avoid actively fearful situations. However, compared to Dunsmoor et al. (2014), our operant conditioning paradigm is a new SFC with a human sample, which is closer to the real learning processes.

Though, some limitations should be taken into account for the current study. Firstly, we are careful generalizing our results to a broader population in view of the high proportion of female psychology students in our sample. However, as social phobia is twice as prevalent in women as in men (Bandelow and Wedekind, 2014), this suggests females an interesting target group for our paradigm. Further studies should consider an only female participant group. Secondly, our physiological results were only partly in concordance with the subjective data. Yet it is not unusual to find an attenuated physiological change following conditioning. First of all, this may be because of the high interindividual differences in fear responses on a physiological level (Craske et al., 1991). It is also possible, that the air blast and scream were not aversive enough to invoke fear conditioning that reflects on the level of physiological measures in all participants. For further research, it should be considered to use electric shocks as US in order to generate more robust fear responses (see Schmitz and Grillon, 2012). In addition, it is important to mention that the FOV of the HMD we used was relatively narrow (diagonal FOV 80°). For further research, we suggest to use other HMDs with a larger FOV to maximize the immersive VR experience. Finally, it should be noted that there was no balancing of clothes and haircut/color within neither CS+, nor CS–. As a consequence it is possible that the learned connection between social stimuli and aversive effects was not predominantly social, but could have been attributable, for example, to the color of the clothes of the agent. Importantly, it is possible that our results are not specific to the social interaction but would have been found also using non-social situations. Nevertheless, a first hint for the specificity is, that we found differences in conditioning between higher and lower socially fearful participants in our study. Further studies could directly investigate the specificity of the paradigm for social situations by manipulating the intensity of the social interaction between the agent and the participant and measuring the effect on conditioning or interactions between social fear and the manipulation within the paradigm. Furthermore, we believe that our paradigm provides the opportunity for a social interaction between the agent and the participant (via eye contact, self-regulated moving of the avatar and movement of the agent). In addition, using the same medium (VR for the context and agent) provides a more flowing and immersive VR experience, which should lead to higher presence and increased emotional responses.

Further research is required to confirm the specificity of our results for social anxiety and transfer the results to social-phobic patients. Also, it is essential to collect data from a general population sample to expand generalizability. Another step would be to compare social-phobic patients and a healthy control group to investigate differences, especially regarding discrimination learning. Furthermore, it is relevant to elicit whether there is dissimilarity between fear reactions in male and female participants. And, accordingly, the general gender influence of social stimuli should be investigated. For further research it would also be interesting to collect data on the time the participants required to approach each of the agents to investigate social fear related avoidance behavior. There are a few improvements that could be applied to our paradigm in order to increase its validity. On the technological level, e.g., one could use more elaborated social agents. In addition, it would be interesting to investigate whether the use of electric shock (a well-established US in fear conditioning), or a more ecological valid US like negative social reactions are more effective than an air blast. Furthermore, one might consider increasing the degree of social interaction, for example, by adding some sort of communication between the agent and avatar. Finally, we would suggest the integration of Heart Rate Variability as a dependent variable in further research as there seems to be a growing body of research indicating its value in fear conditioning (e.g., Pappens et al., 2014) and its relevance to social fear (e.g., Alvares et al., 2013).

In summary, operant conditioning mechanisms work for social stimuli in healthy humans using a VR environment where people actively control their movements. Interestingly, there are hints that higher socially fearful participants generalize aversive learning to unknown persons to a higher extent than lower socially fearful persons and to a less extend differentiate between aversive and non-aversive persons. This VR SFC paradigm seems to be useful in learning and unlearning social fears and could be implemented for further investigations of social anxiety. Our SFC paradigm is a promising first step in establishing a paradigm to acquire a deeper understanding of the underlying mechanisms of development and maintenance of social anxiety (e.g., the involvement of the oxytocin system). On the long term this paradigm may contribute to an efficient therapy (extinction) module for a successful treatment of social phobia. Our SFC paradigm is a promising first step to simulate social fear and its extinction in VR. This could offer a platform to investigate underlying mechanisms of development and maintenance of social fear and has the potential to accelerate the development of more efficient treatments for social phobia.

## Author Contributions

YS, study conception, data analysis, wrote the manuscript. JR, study conception, data acquisition and analysis, and contribution to the manuscript. IN, study conception and contribution to the manuscript. AM, study conception, data analysis, and contribution to the manuscript. All authors have approved the final version of the manuscript and its submission.

### Conflict of Interest Statement

AM is stakeholder of a commercial company that develops virtual environment research systems. YS, JR, and IN have no conflicts of interest.
